# Severity and mortality of COVID 19 in patients with diabetes, hypertension and cardiovascular disease: a meta-analysis

**DOI:** 10.1186/s13098-020-00586-4

**Published:** 2020-08-31

**Authors:** Bianca de Almeida-Pititto, Patrícia M. Dualib, Lenita Zajdenverg, Joana Rodrigues Dantas, Filipe Dias de Souza, Melanie Rodacki, Marcello Casaccia Bertoluci, Brazilian Diabetes Society Study Group (SBD)

**Affiliations:** 1 Departamento de Medicina Preventiva, Escola Paulista de Medicina, Universidade Federal de São Paulo, Rua Botucatu, n° 740, Vila Clementino, São Paulo, SP CEP 04023-062 Brazil; 2 Sociedade Brasileira de Diabetes–SBD, Rua Afonso Braz, 579, Salas 72/74, Vila Nova Conceição, São Paulo, SP CEP 04511-011 Brazil; 3 Programa de Pós-Graduação Em Endocrinologia E Metabologia, Escola Paulista de Medicina, Universidade Federal de São Paulo, Rua Estado de Israel, n° 639, Vila Clementino, São Paulo, SP CEP 04022-001 Brazil; 4 Departamento de Medicina, Escola Paulista de Medicina, Universidade Federal de São Paulo, Rua Sena Madureira, 1500, Vila Clementino, São Paulo, SP CEP 04021-001 Brazil; 5 Departamento de Clínica Médica, Serviço de Nutrologia, Hospital Universitário Clementino Fraga Filho, Universidade Federal Do Rio de Janeiro, Rua Professor Rodolpho Paulo Rocco, 255/sala 9E14, Cidade Universitária, Rio de Janeiro, RJ CEP 21941-590 Brazil; 6 Departamento de Medicina Interna, Faculdade de Medicina, Universidade Federal Do Rio Grande Do Sul (UFRGS), Rua Ramiro Barcelos, 2350, 4º Andar, Porto Alegre, RS CEP 90035‑007 Brazil; 7 Unidade de Endocrinologia, Hospital de Clínicas de Porto Alegre (HCPA-UFRGS), Rua Ramiro Barcelos, 2350, 4º Andar, Porto Alegre, RS CEP 90035‑007 Brazil

**Keywords:** Diabetes, Hypertension, Cardiovascular disease, COVID-19, SARS-CoV-2, Severity, Mortality

## Abstract

**Background:**

The aim of this study is to evaluate the impact of diabetes, hypertension, cardiovascular disease and the use of angiotensin converting enzyme inhibitors/angiotensin II receptor blockers (ACEI/ARB) with severity (invasive mechanical ventilation or intensive care unit admission or O2 saturation < 90%) and mortality of COVID-19 cases.

**Methods:**

Systematic review of the PubMed, Cochrane Library and SciELO databases was performed to identify relevant articles published from December 2019 to 6th May 2020. Forty articles were included involving 18.012 COVID-19 patients.

**Results:**

The random-effect meta-analysis showed that diabetes mellitus and hypertension were moderately associated respectively with severity and mortality for COVID-19: Diabetes [OR 2.35 95% CI 1.80–3.06 and OR 2.50 95% CI 1.74–3.59] Hypertension: [OR 2.98 95% CI 2.37–3.75 and OR 2.88 (2.22–3.74)]. Cardiovascular disease was strongly associated with both severity and mortality, respectively [OR 4.02 (2.76–5.86) and OR 6.34 (3.71–10.84)]. On the contrary, the use of ACEI/ARB, was not associate with severity of COVID-19.

**Conclusion:**

In conclusion, diabetes, hypertension and especially cardiovascular disease, are important risk factors for severity and mortality in COVID-19 infected people and are targets that must be intensively addressed in the management of this infection.

## Introduction

The World Health Organization declared the SARS-CoV-2 outbreak, the Coronavirus Disease–COVID-19, as pandemic on 11th of March of 2020. At that time, it affected 114 different countries, being responsible for about 4 thousand deaths around the world. Until the end of May, SARS-CoV-2 infection had already affected more than 200 countries/regions and accounted for more than 360 thousand deaths worldwide [1].

Considering the prognosis of the COVID-19, approximately 80% of patients have mild illness, 14% have severe illness and 5% critical illness [2, 3]. Current studies and reviews have helped to clarify the clinical profile of COVID-19 [4], regarding to the most prominent symptoms, as well as to factors associated with greater susceptibility to infection and severity of the disease. The initial symptoms do not seem to differ by the presence of comorbidities and the mild clinical presentation of cases was similar in different countries and at different ages [4, 5]. Despite that, studies have shown a worse prognosis for individuals with chronic diseases, such as diabetes (DM), hypertension (HT) or cardiovascular disease (CVD) [4, 5].

Since the first reports of COVID-19 in Wuhan, China, high frequencies of diabetes, hypertension and cardiovascular disease among hospitalized patients and in those with fatal outcome have shown the importance of these co-morbidities as a risk factor for serious outcomes and lethality [6]. Moreover, medications’ mechanism arise as some explanatory hypothesis emerges for this association. It was documented that angiotensin-converting enzyme 2 (ACE2) receptor expression is increased in individuals with diabetes. It has been also hypothesized that some medications could increase ACE2 expression, such as ACE inhibitors and angiotensin 2 receptor blockers (ARBs) that might result in worsening infectivity and severity of SARS-CoV-2 infection due to increased binding to their target cells through ACE2 [7]. However, until now, the evidence has been controversial, as potential adverse effects of these agents on the prognosis of individuals with COVID-19 have not been confirmed.

As the pandemic advanced to other continents, data from Europe and Americas have confirmed the disturbing relationship between diabetes, hypertension and cardiovascular disease and COVID-19 outcomes [8, 9]. These findings represent a worrying public health problem worldwide, when we face the actual burden of non-communicable chronic diseases (NCDs), especially diabetes, hypertension and cardiovascular disease [10]. It is relevant to notice that NCDs accounted for 79.5% of total years lived with disability (YLDs) in 2017, with a total of 678 million YLDs for NCDs causes. In 2019, the International Diabetes Federation (IDF) estimated that 8.8% of the world population between 20 and 79 years old were living with diabetes, representing 415 million people [11]. Considering this scenario, it is critical to better define the precise strength of the association between cardiovascular disease, diabetes and hypertension, with the prognosis of COVID-19, which might help to create more effective prevention strategies in the population. The aim of this study is to evaluate the association of diabetes, hypertension, cardiovascular disease and ACEI/ARBs exposure with severity–intensive care unit treatment or mechanical ventilation necessity or O2 saturation < 90%–or mortality from COVID-19 infection.

## Material and methods

### Systematic literature search

We conducted this study in accordance with the PRISMA Checklist (Preferred Reporting Items for Systematic Reviews and Meta-Analyses) [12] and it was registered in the International Prospective Register of Systematic Reviews (PROSPERO, registration number is CRD42020184254).

A comprehensive search of the PubMed, Cochrane Library and SciELO databases was performed to identify relevant articles published in English and Chinese from December 2019 to 6th May 2020. The search terms were as follows: “ COVID19 disease-CoV-2 infection” (COVID-19 virus disease OR 2019 novel coronavirus infection OR 2019-nCoV infection OR coronavirus disease 2019 OR coronavirus disease-19 OR 2019-nCoV disease OR COVID-19 virus infection) AND severity outcomes terms, that includes “Intensive Care Unit” (Intensive Care Units OR Care Unit, Intensive OR Care Units, Intensive OR Unit, Intensive Care OR Units, Intensive Care), “Need for Mechanical Ventilation” (Respiration, Artificial OR Artificial Respiration OR Artificial Respirations OR Respirations, Artificial OR Ventilation, Mechanical OR Mechanical Ventilations OR Ventilations, Mechanical OR Mechanical Ventilation) or “Death” (Fatal Outcome OR Fatal Outcomes OR Outcome, Fatal OR Outcomes, Fatal OR Determination of Death OR Near-Death Experience OR Cardiac Death OR Death, Cardiac) AND Comorbidities Including “Hypertension and cardiovascular disease” (Blood Pressure, High OR Blood Pressures, High OR High Blood Pressure OR High Blood Pressure OR Vascular Resistance OR Resistance, Vascular OR Total Peripheral Resistance OR Peripheral Resistance, Total OR Resistance, Total Peripheral OR Systemic Vascular Resistance OR Resistance, Systemic Vascular OR Vascular Resistance, Systemic OR Peripheral Resistance OR Resistance, Peripheral), “Diabetes Mellitus” (Glucose Intolerance OR Glucose Intolerances OR Intolerance, Glucose OR Intolerances, Glucose OR Blood Glucose OR Blood Sugar OR Sugar, Blood OR Glucose, Blood OR Hyperglycemia OR Hyperglycemias OR Hyperglycemia, Postprandial OR Hyperglycemias, Postprandial OR Postprandial Hyperglycemias OR Postprandial Hyperglycemia OR Metabolic Cardiovascular Syndrome OR Cardiovascular Syndrome, Metabolic OR Cardiovascular Syndromes, Metabolic OR Syndrome, Metabolic Cardiovascular OR Insulin Resistance). Additionally, references cited in the retrieved articles were also examined to find relevant studies that had not been identified by database searches.

### Inclusion criteria

**Fig. 1:**
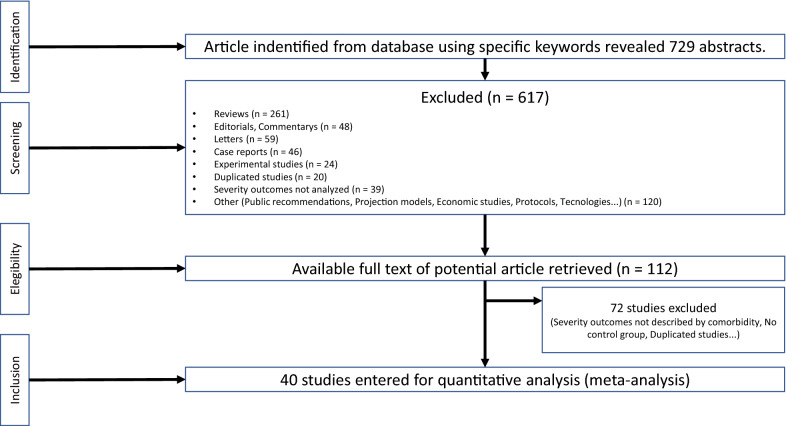
Flow chart (PRISMA) showing the meta-analysis studies selection

Observational studies that met the following criteria were included: (1) study design (cross-sectional, self-controlled case series or retrospective cohort studies); (2) presence of the following comorbidities (diabetes mellitus, hypertension or cardiovascular disease); (3) use of ACE inhibitors and/or ARB. Only adult patients aged 18 years or older were included. We excluded reviews, case report, editorials, letters, expert opinions, animal and experimental studies. We also did not include studies that did not report the interest outcomes (severity or mortality) (see below). Articles in English and Chinese were included. The inclusion process followed the PRISMA flow-chart and is depicted in detail in Fig. 1.

### Outcomes

The primary outcomes were (1) Severity of COVID-19 including: (Intensive Care Unit (ICU) admission or need for mechanical ventilation or low O2 saturation (< 90%) and (2) mortality due to confirmed COVID-19. Only intra-hospital mortality was considered.

### Data extraction and quality assessment

The systematic search was performed by 3 pairs of reviewers who extracted data in a double-blinded manner. Disagreements were solved by discussion within the pair of delegated authors and then confirmed by the whole group.

Data were extracted from the 40 studies selected and recorded using standardized forms. It included authors’ first names, study location, type of study, numbers of participants, the exposition factors (existence of co-morbidities: hypertension, diabetes, cardiovascular disease, use of ACEI/ARB) and the outcomes (Table 1). The quality assessment was performed using the checklist of JBI (Joanna Briggs Institute) Critical Appraisal Tools, for the different type of study included: *Critical Appraisal Checklist for Cohort Studies and Critical Appraisal Checklist for Analytical Cross Sectional Studies* [54].

**Table 1:** Characteristics of studies included in meta-analysis

Reference numbers	First author	Type of study	Location	Sample size	Morbidities (HAS/DM/DCV/ACE OU BRA)	Outcome (severity and/or mortality)
[14]	Li et al.	Case Series	CHINA	362	HAS/DM/DCV/ACEI/ARB uses	Severity and Mortality
[15]	Giamarellos-Bourboulis et al.	Case Series	GREECE	54	DM/DCV	Severity
[16]	Zhang et al.	Retrospective Cohort	CHINA	19	HAS/DM/DCV	Mortality
[17]	Zhou et al.	Retrospective Cohort	CHINA	191	HAS/DM/DCV	Mortality
[18]	Colombi et al.	Retrospective Cohort	ITALY	236	DM/DCV	Severity
[19]	Li et al.	Cross Sectional	CHINA	548	HAS/DM/DCV/ACEI/ARB use	Severity
[20]	Lei et al.	Retrospective Cohort	CHINA	34	DM/DCV	Severity
[21]	Deng et al.	Retrospective Cohort	CHINA	112	HAS/DM/DCV	Severity
[22]	Guo et al.	Case Series	CHINA	187	HAS/DM/DCV/ACEI/ARB use	Severity
[23]	Huang et al.	Retrospective Cohort	CHINA	41	HAS/DM/DCV	Severity
[24]	Zhang et al.	Case Series	CHINA	140	HAS/DM/DCV	Severity
[25]	Liu et al.	Retrospective Cohort	CHINA	78	HAS/DM/DCV	Severity
[26]	Wang et al.	Retrospective Cohort	CHINA	138	HAS/DM/DCV	Severity
[27]	Guan et al.	Retrospective Cohort	CHINA	1099	HAS/DM/DCV	Severity and Mortality
[28]	Wu et al.	Retrospective Cohort	CHINA	84	HAS/DM/DCV	Mortality
[29]	Zhang et al.	Retrospective Cohort	CHINA	221	HAS/DM/DCV	Severity
[30]	Chen et al.	Retrospective Cohort	CHINA	274	HAS/DM/DCV	Mortality
[31]	Deng et al.	Retrospective Cohort	CHINA	225	HAS/DM/DCV	Mortality
[32]	Wang et al.	Case Series	CHINA	69	HAS/DM/DCV	Severity
[33]	Yang et al.	Retrospective Cohort	CHINA	52	DM/DCV	Mortality
[34]	Guan et al.	Case Series	CHINA	1590	DM/DCV	Mortality
[35]	Zheng et al.	Retrospective Cohort	CHINA	161	HAS/DM/DCV	Severity
[36]	Fan et al.	Retrospective Cohort	CHINA	21	HAS/DM/DCV	Mortality
[37]	Yuan et al.	Case Series	CHINA	27	HAS/DM/DCV	Severity
[38]	Feng et al.	Retrospective Cohort	CHINA	476	HAS/DM/DCV	Severity
[39]	Mao et al.	Case Series	CHINA	214	HAS/DM/DCV	Severity
[40]	Wang et al.	Retrospective Cohort	CHINA	339	HAS/DM/DCV	Mortality
[41]	Simonet et al.	Retrospective Cohort	FRANCE	124	HAS/DM/DCV	Severity
[42]	Chen et al.	Cross Sectional	CHINA	150	HAS	Severity
[43]	Wu et al.	Retrospective Cohort	CHINA	201	HAS	Severity
[44]	Xiang et al.	Cross Sectional	CHINA	49	HAS	Severity
[45]	Wan et al.	Cross Sectional	CHINA	135	HAS/DM/DCV	Severity
[46]	Qin et al.	Retrospective Cohort	CHINA	452	HAS	Severity
[47]	Ruan et al.	Retrospective Cohort	CHINA	150	HAS	Mortality
[48]	Zhang et al.	Retrospective Cohort	CHINA	1128	ACEI/ARB use	Mortality
[49]	Xu et al.	Retrospective Cohort	CHINA	187	HAS	Mortality
[50]	Meng et al.	Case series	CHINA	42	ACEI/ARB use	Severity
[51]	Yang et al.	Retrospective Cohort	CHINA	125	ACEI/ARB use	Severity
[52]	Mancia et al.	Retrospective Cohort	ITALY	6272	ACEI/ARB use	Severity
[53]	Reynolds et al.	Retrospective Cohort	USA	2005	ACEI/ARB use	Severity

DM, diabetes mellitus; HAS, hypertension; DCV, cardiovascular disease; ACEI/ARB, angiotensin converting enzyme inhibitors/angiotensin II receptor blockers

### Data synthesis and analysis

All meta-analytical calculations were performed with Comprehensive Meta-Analysis Software (CMA) version 3.0. To provide a quantitative estimate of the association of the morbidities of interest (diabetes, hypertension and cardiovascular disease) with severity outcomes (ICU admission or need for mechanical ventilation) or death in COVID-19 patients, adjusted ORs with corresponding 95% confidence intervals (CIs) (Table 1) were calculated from crude frequency of exposed and non-exposed cases. Statistical heterogeneity was estimated by χ^2^ test and the I^2^ statistic. A p value < 0.05 for the Q-statistic was considered to indicate substantial heterogeneity. Random-effects model was used for comparison to address heterogeneity. To assess publication bias, through the qualitative assessment, we performed Funnel Plots and evaluated the distribution visually and by performing the Egger’s regression test (Additional file 1) [13]. Statistical significance was set at a level of p < 0.05.

## Results

### Search results

The initial search strategy identified 729 studies, and 617 were excluded after reading title and abstract, because of excluding criteria, including duplicated studies and lack of specified outcomes. The remaining 112 articles underwent full-text-evaluation. This phase retrieved 40 articles which were included in the meta-analysis. This involved 18.012 COVID-19 patients from 27 retrospective cohorts, 9 case-series and 4 cross-sectionals studies. The studies characteristics are shown in Table 1. Thirty-five studies were performed in China and 5 were from France, Italy, Greece, and United States of America. All selected studies were published in 2020. In 25 studies clinical outcomes were exclusively severity, in 12 studies the only outcome was mortality and 2 studies evaluated both “Severity” and “Mortality”.

### Meta-analysis

#### Diabetes mellitus

**Fig. 2:**
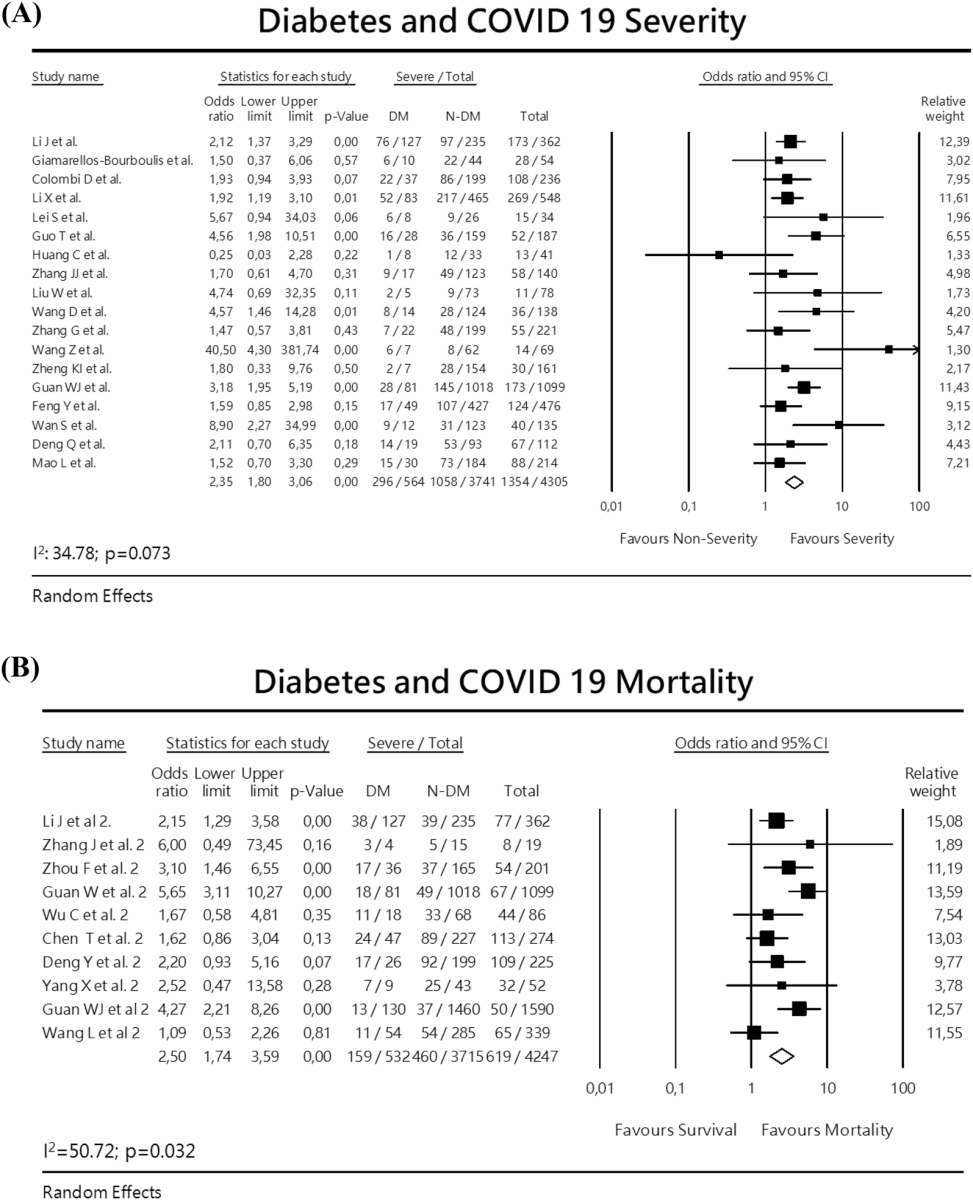

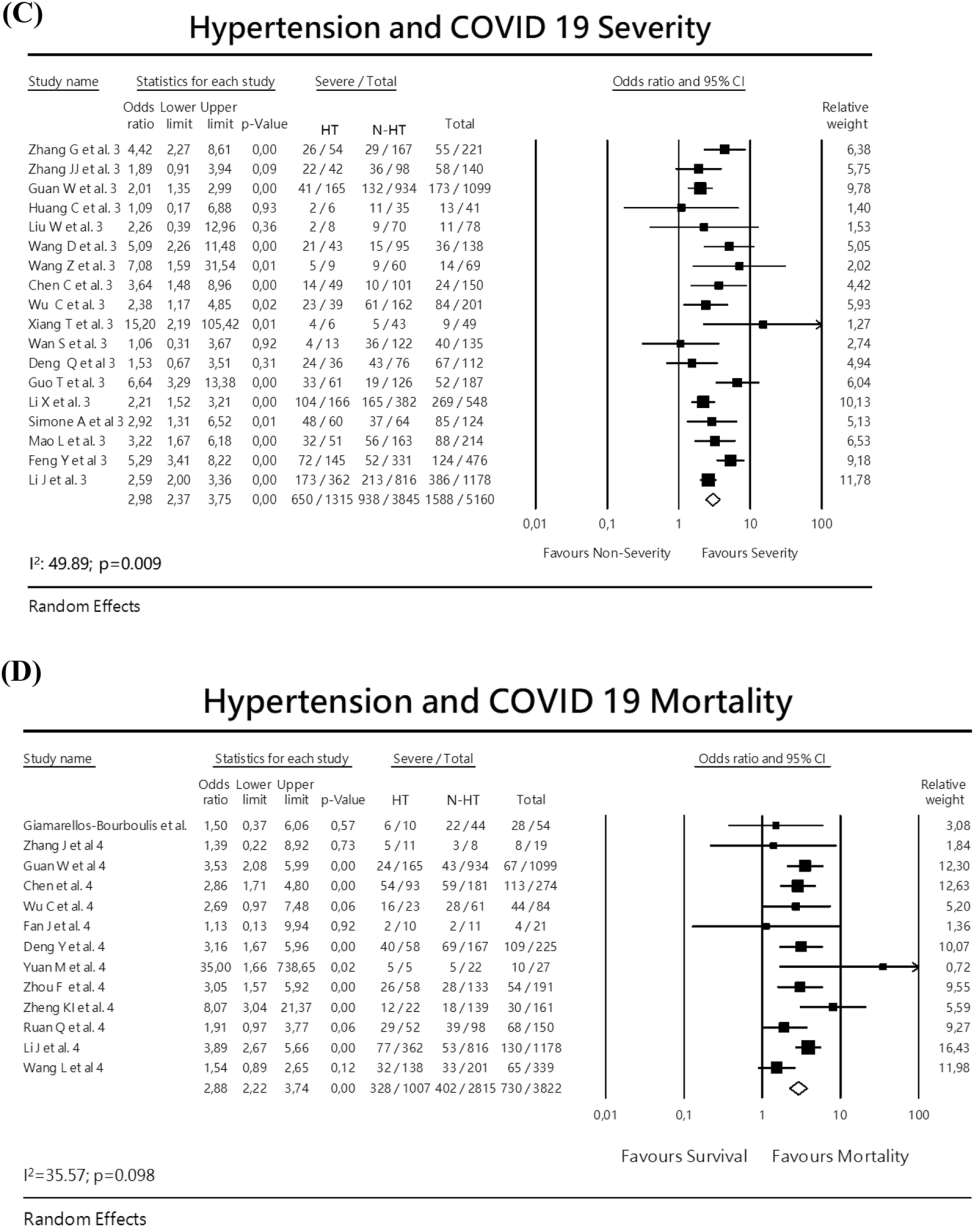

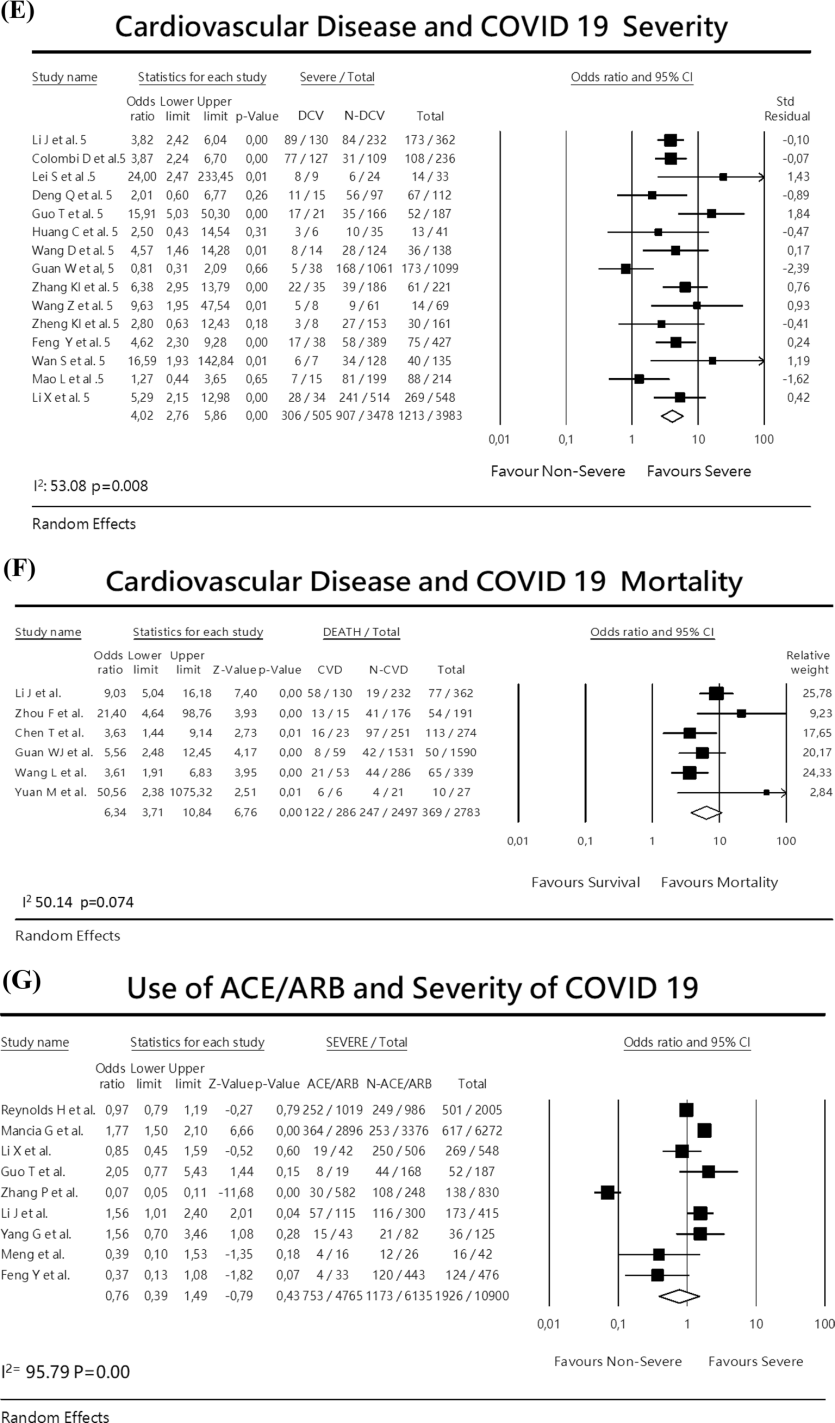
Meta-analysis with forest plot presenting the OR and 95% CI for severity or mortality of COVID-19 according to the presence of diabetes mellitus, hypertension, cardiovascular disease or ACEI/ARB exposure. **a** Diabetes and COVID-19 severity, **b** Diabetes and COVID-19 mortality, **c** Hypertension and COVID-19 severity, **d** Hypertension and COVID-19 mortality, **e** Cardiovascular disease and COVID-19 severity, **f** Cardiovascular disease and COVID-19 mortality, **g** ACEI/ARBs exposure and COVID-19 severity

For the analysis of severity, 18 studies were included, with a total of 4.305 patients with 564 diabetes analyzed. A random-effect meta-analysis estimated a pooled odds-ratio (OR) of 2.35 (95% CI 1.80–3.06) (Fig. 2a). In this analysis, there was mild heterogeneity (I^2^ = 34.78; p = 0.073), with no significant publication bias (Eggers test intercept: 0.5817, p = 0.37).

For the analysis of mortality, 10 studies were included (8 retrospective cohort and 2 case-series studies). A random-effects analysis showed a pooled OR of 2.50 (95% CI 1.74–3.59). There was a moderate heterogeneity I^2^ 50.72 p = 0.032. No publication bias was detected. Eggers test intercept was – 0.0397; p = 0.977. (Fig. 2b).

### Hypertension

The association of hypertension and severity of COVID-19 18 studies were analyzed, including a total of 5160 patients, being 1315 with hypertension. The random-effects meta-analysis calculated an OR 2.98 (95% CI 2.37–3.75) (Fig. 2c) There was a moderate heterogeneity I^2^ = 49.89 p = 0.009. There was no publication bias: Eggers test: – 0.4887; p = 0.48. In the analysis of mortality, 13 studies were included, with a total of 3.822 patients, being 1007 with hypertension. The random-effect analysis indicated an OR 2.88 (95% CI 2.22–3.74) (Fig. 2d). In this analysis, there was no publication bias: Eggers test – 0.20021; p = 0.80.

### Cardiovascular disease

The association with COVID-19 severity was analyzed in 15 studies, enrolling a total of 3983 patients being 505 with previous history of cardiovascular disease. The random-effects analysis lead to an OR of 4.02 (95% CI 2.76–5.86) (Fig. 2e). There was no publication bias. Eggers test 0.49655 p = 0.59. The analysis considering mortality due to COVID-19 retrieved 6 studies evaluating 2783 individuals being 286 with cardiovascular disease. The random-effects analysis resulted a pooled OR of 6.34 (95% CI 3.71–10.84) (Fig. 2f).

### ACEI/ARB use

For this analysis, 9 studies were included, with a total of 10.900 COVID-19 cases, being 4.765 on ACEI/ARB. The random-effects analysis showed a combined OR for severity 0.76 (95% CI 0.39–1.49) (Fig. 2g). There was a high heterogeneity I2:95.79, p = 0.00. This indicated that there was no association between use of ACEI/ARB and COVID-19 severity.

## Discussion

This is one of the largest meta-analysis, to our knowledge, associating cardiovascular disease, diabetes and hypertension with COVID-19 outcomes. It was able to analyze 40 papers, with 18.012 confirmed COVID-19 patients, including 564 with diabetes, 1315 with hypertension and 505 with cardiovascular disease. We identified a moderate positive association between diabetes mellitus and hypertension with COVID-19 severity and mortality, and a strong positive association with the report of previous cardiovascular disease with both outcomes. On the other hand, no association was found between the use of ACE/ARBs with severity.

The link between diabetes and COVID-19 outcomes was previously detected in meta-analyses with a smaller number of studies [55–58]. In a previous meta-analysis from our group [59], we analyzed 7 studies, including 1.592 confirmed COVID-19 patients, being 138 with diabetes. We showed that diabetes is an important risk factor for COVID-19 severity [OR: 3.53 (1.48–8.39)]. In that study there was a high heterogeneity that was addressed by random-effect analysis and meta-regression, using the mean age as a covariate, with no impact on results, indicating that diabetes risk was independent of age. In the present study, we identified a 2.3-fold increase in the risk of severity and a 2.5-fold increase for mortality associated with COVID-19 in patients with DM. Our results are similar to data reported by Kumar et al., who showed an OR of 2.75 (95% CI 2.09–3.62) for the association of diabetes with severity and an OR of 1.90 (95% CI 1.37–2.64 for diabetes and mortality [60]. Other recent meta-analysis including approximately 30 studies each (16.003 and 6.452 patients, respectively) were performed aiming to investigate the association between solely diabetes with severity and mortality of COVID-19, but the criteria to define disease severity were more variable than those defined in the present study. The authors found that diabetes was associated with mortality (RR 2.12 [1.44, 3.11], p < 0.001; I^2^: 72%) and severity of the COVID-19 infection (RR 2.45 [1.79, 3.35], p < 0.001; I^2^: 45%) [61]. Patients with diabetes also have a more unfavorable outcome in others common infections such as Severe Acute Respiratory Syndrome (SARS-CoV) and Middle East Respiratory Syndrome (MERS-Cov) as was described in previous out brakes [62, 63].

Multiple pathophysiological mechanisms can support the association between DM and COVID-19 severity; however, much of knowledge is derived from SARS-COV infection rather than COVID-19. Compromised innate immune system due to chronic hyperglycemia, pro-inflammatory state characterized by inappropriate and exaggerated cytokine response and underlying pro-thrombotic hypercoagulable have been implicated in this association [62, 63].

Regarding hypertension, Pranata et al. have performed a meta-analysis, searching for the association including solely hypertension and COVID-19 severity/mortality, with 6.560 patients from 30 studies published in PUBMED and in other databases. The authors found that a diagnosis of hypertension was associated with increased mortality (RR 2.21 (1.74, 2.81), p < 0.001) and COVID-19 severity (RR 2.04 (1.69, 2.47), p < 0.001) [64]. However, the association between hypertension and worse outcomes of COVID-19 infection may be due to the higher frequency of comorbidities and a more advanced age of these individuals. An Italian cross-sectional study did not find hypertension as an independent factor affecting the outcome of COVID-19 [65].

We found that the highest effect size for severity and mortality was related to cardiovascular disease (which comprised atherosclerotic cardiovascular disease in most studies and included myocardial infarction, stroke and/or peripheral arterial disease). This corresponds about the double of the risk attributed to diabetes and hypertension for mortality alone, indicating that pre-existing cardiovascular disease is the greatest risk factor to be addressed. Although cardiovascular disease was recognized as an important risk factor for these outcomes in most studies, the intensity of association is variable, possibly due to different definitions and severity of cardiovascular disease. While some authors included cerebrovascular diseases, others included only those with coronary heart disease and/or heart failure.

The mechanism associated for the development of atherosclerotic cardiovascular disease is complex and involves a pathological process associated with oxidative stress, inflammation and a prothrombotic status [66]. These same mechanisms that cause damage and vascular remodeling are observed in individuals with type 2 diabetes, obesity and hypertension [65]. Infections with other species of coronaviruses such as SARS-CoV and MERS-COV also pointed to an increased risk of mortality in patients with cardiovascular disease [62, 63]. Initial reports indicated similar outcome with the novel coronavirus [55, 56]. The mechanism implicated in the association between COVID-19 severity or mortality and cardiometabolic factors is still under investigation. Potential explanations include the high prevalence of cardiovascular disease in older people (another established risk factor for adverse outcome), a functionally impaired immune system, and an elevated angiotensin converting enzyme-2 (ACE2) receptor expression [66]. Coronaviruses can bind to target cells through angiotensin-converting enzyme 2 (ACE2) receptor which is expressed in several tissues and is involved in the renin–angiotensin–aldosterone system (RAAS) [67]. ACE2 receptor is a homologue of the angiotensin-converting enzyme that converts angiotensin II to angiotensin 1 to 7 and decreases the vasoconstriction mediated by the renin-angiotensin system and, also, the pro-inflammatory role of angiotensin II. The binding of SARS-CoV-2 to ACE2 receptor can result in alteration of its post-receptor signaling pathways, leading to vasoconstriction, pro-inflammatory response and to endothelial dysfunction that could result into myocardial injury and prothrombotic processes [68].

It is possible that mechanism mediated by ACE2 receptor links type 2 diabetes, hypertension and cardiovascular disease with a higher risk of severe manifestations of COVID-19 infection. It was also documented that some medications can increase ACE2 expression, such as ACE inhibitors (ACEI) and angiotensin 2 receptor blockers (ARBs). In this study, however, the association between the use ACE or ARB and severity or mortality of COVID-19 was not conclusive. We found a remarkably high heterogeneity even after random-effects analysis. We identified the source of heterogeneity in the 9 studies included in this analysis. There were 3 studies [38, 48, 50] indicating a protective effect of ACE/ARBs, 2 were neutral [19, 53] and 4 [14, 22, 52, 69] indicated a trend to deleterious effect. This discrepancy may be related to the age of individuals included in these various studies, since in some analysis, the deleterious effect of ACEI and ARBs seemed to occur in those with an older age [52, 70, 71], while in others, the association disappeared after adjustments for age [52]. Further studies are necessary to elucidate the risk association in different age groups.

This study has several limitations. First, it included cross-sectional, retrospective cohort and series of cases, with a lack of prospective studies in the field. Second, most of the studies were performed exclusively in China. As the pandemic is advanced through different countries, more data will become available to understand if these results are also valid for other populations. Third, although we have observed a higher intensity in the association between mortality and severity of COVID-19 with cardiovascular disease in comparison to hypertension and diabetes, all three diseases commonly coexist in the same individuals and it is likely that one might have influenced the risk attributed to the others. As the number of patients with an overlap of these conditions was not reported in most studies, it was not possible to determine the independent risk for each of these factors. Finally, since information was scarce in the studies, we were not able to investigate the interference of age, type of diabetes, glycemic control and obesity, a significant risk factor for COVID-19 severity and/or mortality [71, 72]. The strength of this meta-analysis was the large number of patients evaluated that brought important novel data. It is currently one of the largest meta-analysis to investigate the association between the severity and mortality of COVID-19 and hypertension, diabetes, cardiovascular disease and ACEI/ARBs exposure. It is relevant to notice that, although it has been few months since the pandemic started, our results of association have some important criteria to rely in these associations. One of these is the consistency of the data throughout many studies, which is the main strength in metanalysis in general and so is in our study. In our study, the metanalysis showed Odds Ratio with relevant strength of association (OR was around 2, 00 or more) and statistical significance (by 95% CI and p values), which means that the probability of this association to be by random is very low (less than 5%). Also, the biological plausibility corroborates for this association. Considering all these points together, our results reinforce the relevant association of diabetes, hypertension and cardiovascular disease with severity and mortality in COVID-19.

In conclusion, diabetes, hypertension and especially cardiovascular disease, are important risk factors for severity and mortality in COVID-19 infected people and are targets that must be intensively addressed in the management of COVID-19.

## Supplementary information

**Additional file 1.** Funnel Plot of each of the morbidities and severity or mortality in COVID-19.

## Data Availability

The datasets used and/or analyzed during the current study are available from the corresponding author on reasonable request.

## Abbreviations

ACEI/ARB: angiotensin converting enzyme inibitors/angiotensin II receptor blockers
ACE: angiotensin-converting enzyme
ACE2: angiotensin-converting enzyme 2 receptor
ARB: angiotensin II receptor blocker
CIs: confidence intervals
CMA: Comprehensive Meta-Analysis Software
COVID-19: 2019 novel coronavirus
CVD: cardiovascular disease
DM: diabetes mellitus
ICU: intensive care unit
IDF: International Diabetes Federation
MERS-Cov: Middle East Respiratory Syndrome
NCDs: non-communicable chronic diseases
PRISMA: Preferred Reporting Items for Systematic Reviews and Meta-Analyses
PROSPERO: International Prospective Register of Systematic Reviews
RAAS: renin–angiotensin–aldosterone system
SARS-CoV: Severe Acute Respiratory Syndrome
SBD: Brazilian Diabetes Society/Sociedade Brasileira de Diabetes
YLDs: years lived with disability
