# Supplementary material for: Severity and mortality of COVID 19 in patients with diabetes, hypertension and cardiovascular disease: a meta-analysis
Source: Diabetol Metab Syndr. 2020 Aug 31;12:75. doi: 10.1186/s13098-020-00586-4 (PMC7456786; doi:10.1186/s13098-020-00586-4)

**Additional file**

Funnel Plot of each of the morbidities and severity or mortality in COVID-19

**Diabetes and COVID 19 Severity**

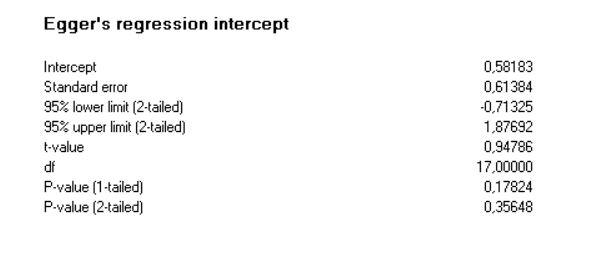


**Diabetes and COVID 19 Mortality**

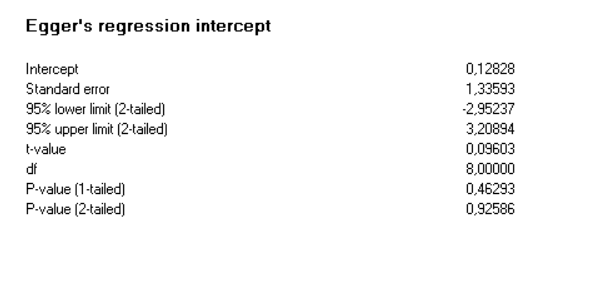


**Hypertension and COVID-19 Severity**

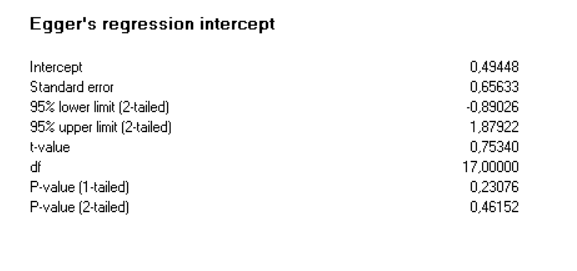


**Hypertension and COVID-19 Mortality**

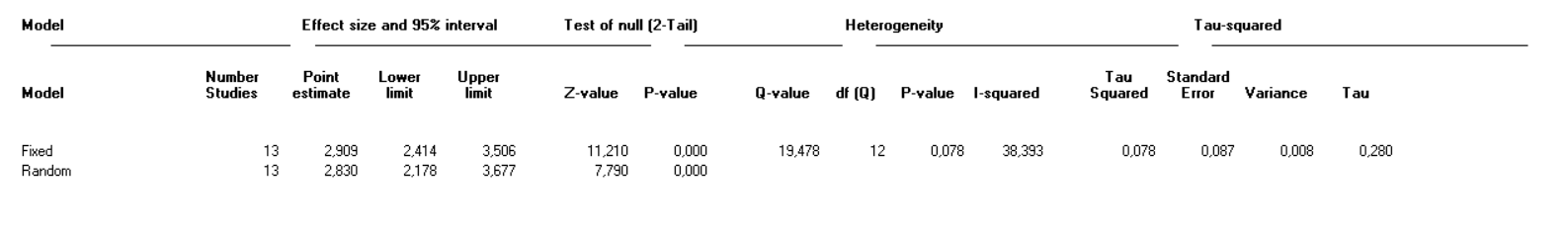


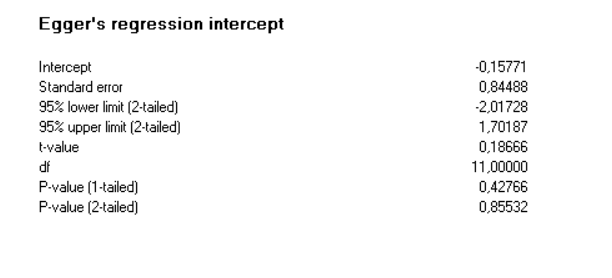


**Cardiovascular Disease and COVID-19 Severity**

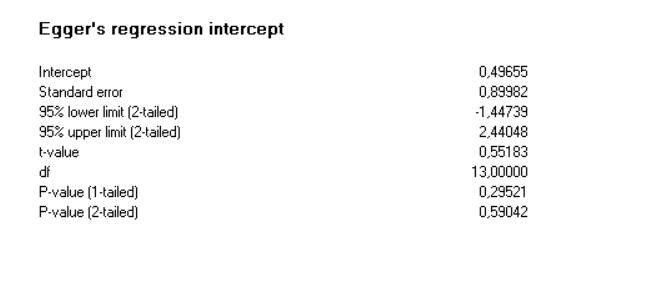


**Cardiovascular Disease and COVID-19 Mortality**

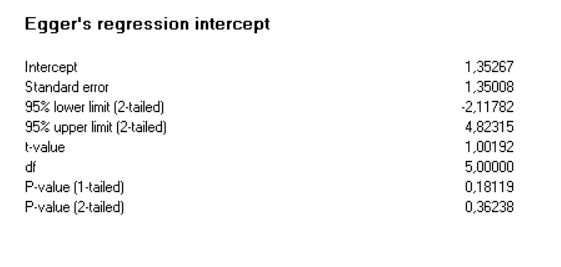


**ACEI/BRAs exposure and COVID-19 Severity**

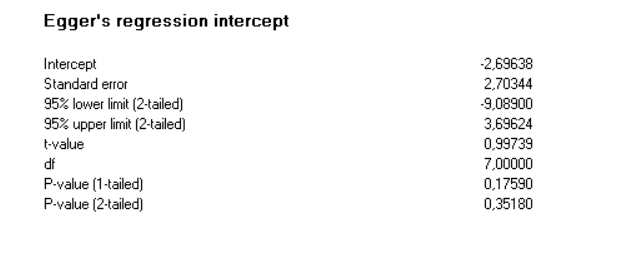

Supplement: Supplementary file 1 — Additional file 1. Funnel Plot of each of the morbidities and severity or mortality in COVID-19. [file 13098_2020_586_MOESM1_ESM.docx]
